# Treating High COD Dyeing Wastewater via a Regenerative Sorption-Oxidation Process Using a Nano-Pored Activated Carbon

**DOI:** 10.3390/ijms23094752

**Published:** 2022-04-26

**Authors:** Shih-Fu Ou, Dun-Sheng Yang, Jia-Wei Liao, Shyi-Tien Chen

**Affiliations:** 1 Department of Mold and Die Engineering, National Kaohsiung University of Science and Technology, Kaohsiung City 80778, Taiwan; m9203510@nkust.edu.tw; 2 Ph.D. Program in Engineering Science and Technology, College of Engineering, National Kaohsiung University of Science and Technology, Kaohsiung City 82445, Taiwan; i108109110@nkust.edu.tw; 3 Innolux Corporation, No. 3, Section 1, Huanxi Road, Xinshi District, Tainan City 74147, Taiwan; lex95100@yahoo.com.tw; 4 Department of Safety, Health and Environmental Engineering, National Kaohsiung University of Science and Technology, Kaohsiung City 82445, Taiwan

**Keywords:** a regenerable adsorption–oxidation process, activated carbon (AC), adsorption capacity, high COD wastewater, hydrogen peroxide

## Abstract

Nowadays, the structural complexity of dyes used in the textile industry and the widely adopted water-saving strategy in the dyeing processes often fail plants’ biological wastewater treatment units due to chemical oxygen demand (COD) overload. To alleviate this problems, this study investigated a regenerable adsorption–oxidation process to treat dyeing wastewater with COD around 10,000 mg/dm^3^ using a highly nano-pored activated carbon (AC) as a COD adsorbent, followed by its regeneration using hydrogen peroxide as an oxidizing reagent. In addition to studying AC’s COD adsorption and oxidation performance, its operational treatment conditions in terms of temperature and pH were assessed. The results firstly demonstrated that about 50–60% of the COD was consistently adsorbed during the repeated adsorption operation before reaching AC’s maximum adsorption capacity (q_max_) of 0.165 g-COD/g-AC. The optimal pH and temperature during adsorption were 4.7 and 25 °C, respectively. Secondly, AC regeneration was accomplished by using an initial peroxide concentration of 2.5% (by wt %) and EDTA-Fe of 2.12 mmole/dm^3^. The reuse of the regenerated ACs was doable. Surprisingly, after the first AC regeneration, the COD adsorption capacity of the regenerated AC even increased by ~7% with respect to the virgin AC. Thirdly, the results of a five-consecutive adsorption–regeneration operation showed that a total of 0.3625 g COD was removed by the 5 g AC used, which was equivalent to an adsorption capacity (q) of 0.0725 (= 0.3625/5) g-COD/g-AC during each adsorption stage. Based on the obtained results, a regenerable COD adsorption–oxidation process using a nano-pored AC to treat the high-textile-COD wastewater looks promising. Thus, a conceptual treatment unit was proposed, and its potential benefits and limitations were addressed.

## 1. Introduction

Diverse types of dyes are commonly used in modern textile dyeing industry [1,2]. In general, different types of dyes such as acid, reactive, and disperse dyes have been developed for use in dyeing animals, plants, and artificial fibers, respectively. Chemical properties of the major dyes vary significantly (Table 1), and some dyes and their derivatives are known carcinogens [3,4,5]. Thus, the hazard and treatability of the residual dyes are of concern. To minimize the risks posed by their mutagenicity, a variety of alternative treatments have been proposed. In particular, biological [6,7,8], physical/chemical [9,10], and oxidative or photo-oxidative [11,12,13,14,15,16,17,18,19] approaches as well as combinations of different methods [20,21,22] have been studied intensively to ensure the chemical oxygen demand (COD) of treated effluent below certain regulatory limits.

The treatment of dyeing wastewater is a great challenge in Taiwan’s dyeing companies nowadays for two reasons: first, the water-saving operations during the dyeing process nearly doubles the influent COD in the dyeing wastewater treatment plant; second, the use of structurally more complex dyes serves various customers’ needs, which increases the difficulty of treating their residues. For example, disperse dyes are fine particulates and have fairly low water solubility, making them difficult to treat. When these less degradable dyes are present in COD-intensive wastewater due to in-plant water-saving operations, the existing activated sludge units are often overloaded, resulting in the failure of the treated effluent to meet the COD regulatory limits. This situation was alarmed by many textile companies in a Textile Media Workshop [23] held by the Southern Taiwan Textile Research Alliance in Tainan City, Taiwan, especially for the companies aiming at a water-reuse and/or zero-waste manufacturing innovation. Honestly, the enlargement of biological units could solve the problem straightforward. However, due to the limited land space in some plants and the high costs in oxygen supply, such a kind of solution is not preferable. On the other hand, some efficient and space-saving alternatives are of interests.

Although various degrees of successes have been achieved by using conventional treatment methods for dye wastewater, the oxidative approach is generally believed as the most efficient. However, Du and Chen [24] concluded that the oxidative removal of disperse dyes (DB-EX-SF) by regular peroxide treatment was minimal. To remove the disperse dye, a relatively high concentration of hydrogen peroxide dosage was suggested [24] but not yet being tested. Other problems such as a huge volume of water used in the dyeing processes that demands a great amount of peroxide make such an oxidative treatment economically undesirable. In addition, a high peroxide residual could halt the performance of the downstream biological units. Thus, we proposed to adsorb high COD out of wastewater first, followed by regenerating COD-saturated activated carbon (AC) for its reuse.

To achieve efficient and stable COD removal at reasonable costs, processing water at 300 m^3^/day from some dyeing companies in Southern Taiwan is divided into two streams: one has a high COD content of around 10,000 mg/dm^3^, and the other has a COD content of around 1000 mg/dm^3^, with a volume ratio around 1:4. Without processing water separation, the influent COD in biological units (i.e., activated sludge basins with one day of hydraulic retention time) is around 2800 mg/dm^3^. The high COD influent results in a low COD removal rate of 75%, causing final COD effluent to be usually around 200–250 mg/dm^3^ and to exceed the regulatory limits of 140 mg/dm^3^. With separation, however, an effective and efficient alternative to treating the high-strength COD flow is urgently needed.

This study proposed to treat the high COD dyeing wastewater in two steps: first, AC was used to adsorb dyes, and second, the adsorbed dyes were treated by a high dosage of hydrogen peroxide. Due to less water with the COD-saturated ACs, the peroxided amount could be significantly reduced. Moreover, precise peroxide dosage could remove the adsorbed COD on the AC surface with a minimal destruction of the AC structure, allowing the reuse of the regenerated AC. To test the applicability of the proposed regenerable adsorption–oxidation treatment, a high-COD influent was collected and used to examine the AC performance. The adsorption capacity of the purchased AC was revealed, so were the operational adsorption conditions of pH and temperature. Next, COD-saturated ACs were oxidatively regenerated by peroxide and an EDTA–Fe complex as an oxidant and an iron catalyst, respectively [24], and the optimal operational conditions were also determined. The precise amount of the peroxide used was considered important and had two major functions: one oxidized the absorbed COD on the ACs without a destruction of the AC structure, and the other broke the complex dye structure into readily biodegradable subunits treated by the following biological treatment. Finally, the percent COD removal was confirmed by mass balance calculations. Upon the completion of the adsorption–oxidation treatment, the aim was to reduce 50% of the COD in the high COD stream. According to mass balance calculations, after its 50% reduction, the two-stream-merged wastewater COD would go down to 1800 mg/dm^3^, which met the design capacity of the existing biological treatment units.

## 2. Results and Discussion

**Figure 1:**
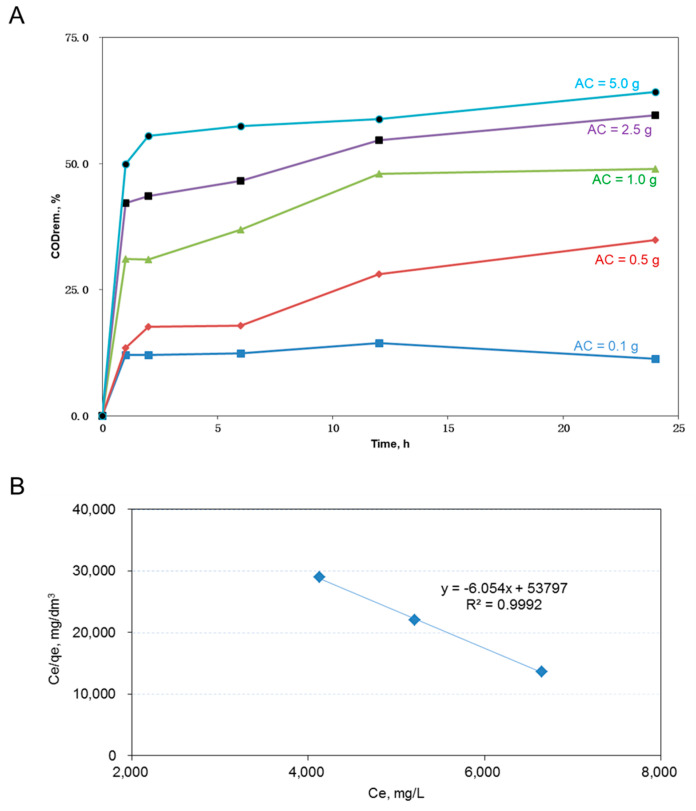
Adsorption characteristics of the used AC. (**A**) Plots of the percent COD removed versus the AC amount used in adsorbing the dyeing wastewater COD. (**B**) Plot of the Ce/qe ratio versus Ce. The maximum adsorption capacity (q_max_) at 24 h in (**A**) was equal to 0.165 (calculated by 1/6.054) g-COD/g-AC.

For AC adsorption, the percent COD removal out of the 60 mL dyeing wastewater using 0.1 to 5 g of AC (i.e., 1.7–83.3 g-AC/dm^3^) are given in Figure 1A. Greater amounts of AC resulted in higher COD removal from wastewater. The highest COD removal reached more than 60% with 5 g of AC, and the achievement of a higher percent removal might not be possible because bigger particulates such as fiber fragments or conglomerates of dyeing chemicals would not be adsorbed by the AC [25]. Figure 1B shows the plot of the final COD concentration (Ce) over the adsorption capacity (qe) versus Ce. Since the linear relationship was fitted with the Langmuir isotherm, the corresponding maximum adsorption capacity (q_max_), which equals one over the slope value of the fitted line, equaled 0.165 (calculated by 1/6.054) g-COD/g-AC). A high R^2^ value of 0.9992 showed a good fit of the employed Langmuir model. These results revealed that using the purchased AC to remove 50% of the tested 60 mL wastewater was doable. A 50% COD reduction would roughly double the hydraulic retention time for the microbes to consume the slowly biodegradable disperse dyes in the activated sludge basins.

**Figure 2:**
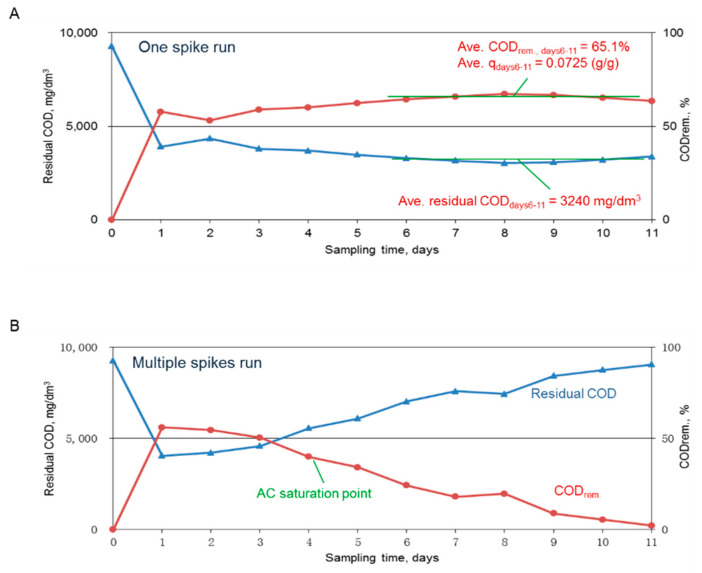
Plots of the residual COD over the sampling time for one spike (**A**) and multiple spikes (**B**) of the dyeing wastewater. For one spike run, the adsorption was nearly completed in one day. For the multiple spikes run, the percent removal of COD dropped to less than 50% on the 4th spike of the wastewater.

The results for the residual COD and the removed COD after using of 5 g of AC to adsorb one spike or multiple spikes of the dyeing wastewater are shown in Figure 2A,B, respectively. Figure 2A reveals that the residual COD for one spike of 60 mL dyeing wastewater leveled off at 3240 mg COD/dm^3^, which was consistent with previous data. The results also revealed that the adsorption reaction was completed within a day. In the contrast run, the same amount of dyeing wastewater was used to replace the treated wastewater daily, and the results revealed that the AC adsorption capacity declined over time and was nearly exhausted by the 11th day. These results indicated that without regeneration the AC could absorb ~65% COD of the tested wastewater; however, via a daily operation, the COD removal rate was maintained at higher than 50% by day 3. Since a 50% or higher COD removal rate was targeted, saturated AC needed to be wasted on day 3 if not being regenerated.

**Figure 3:**
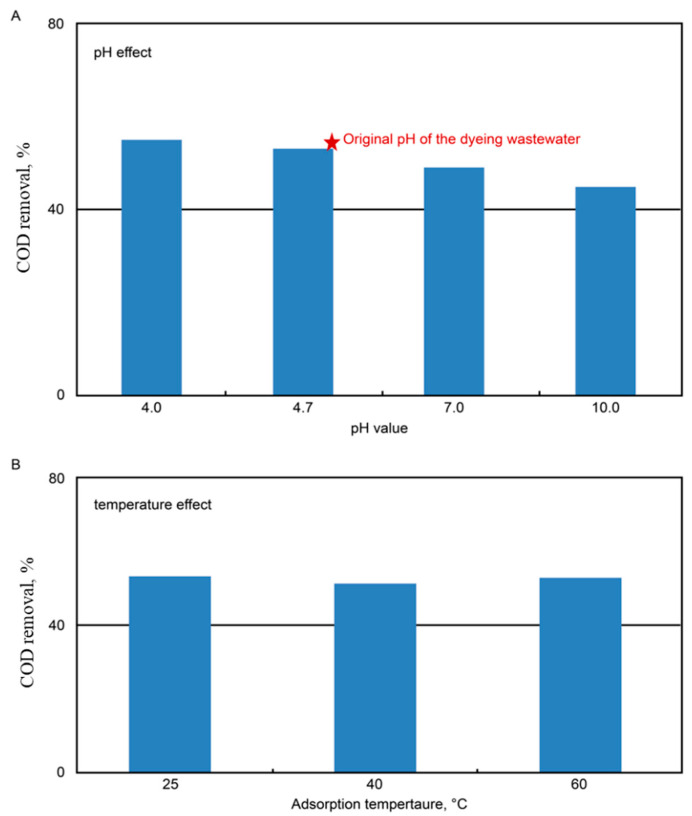
Effects of pH at ambient temperature (**A**) and temperature at pH = 4.7 (**B**) during AC adsorption runs. A lower pH value showed a greater percent COD removal, but the temperature had little effect.

**Figure 4:**
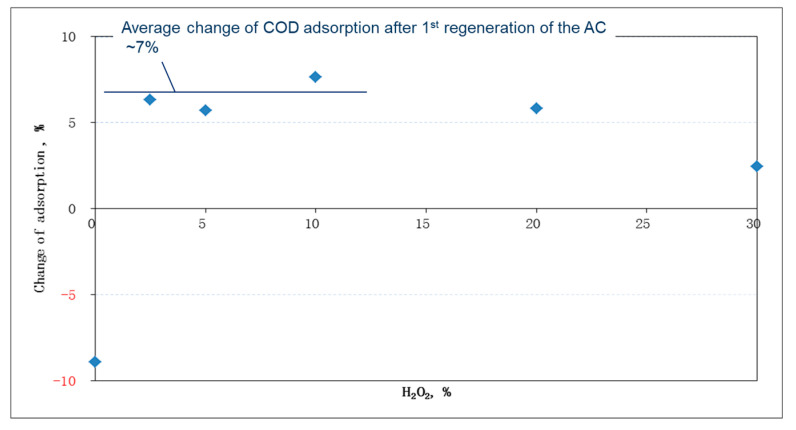
Plots of the changes in the AC adsorption capacity after its first regeneration at various peroxide concentrations and a fixed initial iron-complex content of 2.12 mmole/dm^3^. The results showed that the use of the initial peroxide concentration at 2.5% was sufficient to regenerate the saturated ACs.

The factor analysis during adsorption operations showed that there were adverse effects on COD removal as pH increased over the range of 4–10 (see Figure 3A), which might be caused by the reduced dissociation of dye molecules at acidic conditions. Similar results were concluded by Al-Degs et al. [26]. On the other hand, the temperature effect on adsorption was minimal at 25–60 °C (see Figure 3B). Although individual dye adsorption could be either exothermic or endothermic [26], the use of real wastewater might contain both types of dyes, which leveled off the temperature effect. Practically speaking, the collected data suggested that there was no need to control either pH (since the original pH remained low at 4.7) or temperature during the adsorption stage. The results shown in Figure 4 demonstrated that adsorption capacity increased after treatment by oxidative regeneration. In comparison with the virgin AC, the regenerated AC displayed an increase in adsorption capacity by about 7% on average, if the peroxide dose was less than 10%. On the other hand, the adsorption capacity dropped by nearly 10% in the non-regenerated case (i.e., an initial peroxide content of 0%). Moreover, an increase of the peroxide dose appeared to lower the adsorption percentage of the regenerated AC, especially in the case of a peroxide dose of 30%, which might result from the destruction of the AC pore structure. Thus, a peroxide dose of 2.5% was considered sufficient in regenerating the saturated AC.

The results of the first AC regeneration implied that more dyes (up to 7%) were adsorbed by regenerated AC than by virgin AC. It was reported that the EDTA–Fe complex and peroxide greatly oxidized various dyes [24]. Thus, three hypothetic reasons were proposed to explain the increase of AC adsorption capacity: (1) some debris was virgin AC was oxidized and removed to boost the adsorption capacity of regenerated AC; (2) not only the adsorbed dyes, but also a portion of AC was oxidized and a more pore surface area was gained; and (3) some residue oxidative reagents remained in the AC, resulting in the oxidation of some wastewater COD at the next adsorption. Further investigation on this phenomenon could be helpful to prolong the operational lifetime of the used ACs.

**Figure 5:**
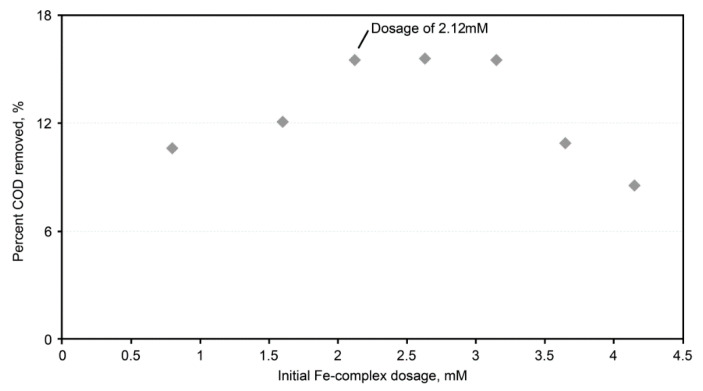
Plots of the percent COD removed after its regeneration at various initial Fe-complex dosages and a peroxide concentration of 2.5%. The use of an initial Fe complex at 2.12 mM had the highest percent COD removal value. A higher Fe complex could consume the oxidative capacity of the peroxide and resulted in the drop of the percent COD removal.

**Figure 6:**
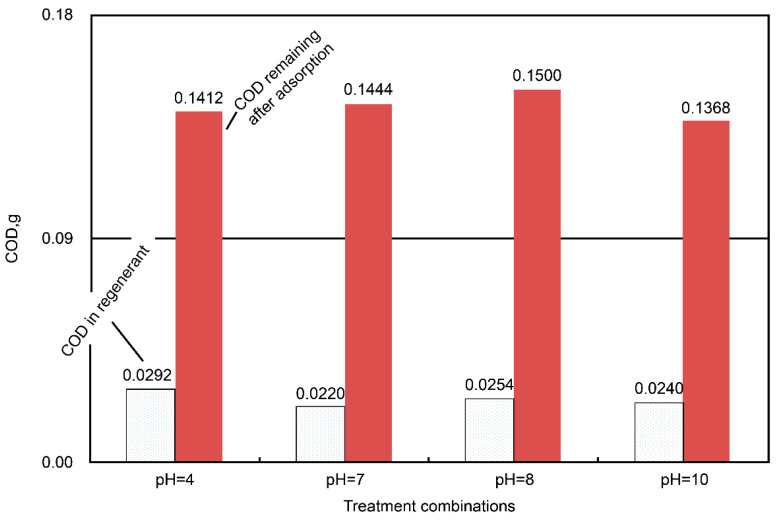
Plots of the COD residues (by weight) in the regenerant and the amounts of the remaining COD in the AC-treated wastewater under various regenerative pH conditions at ambient temperature. The variation of the pH showed little effect on the AC regeneration.

**Figure 7:**
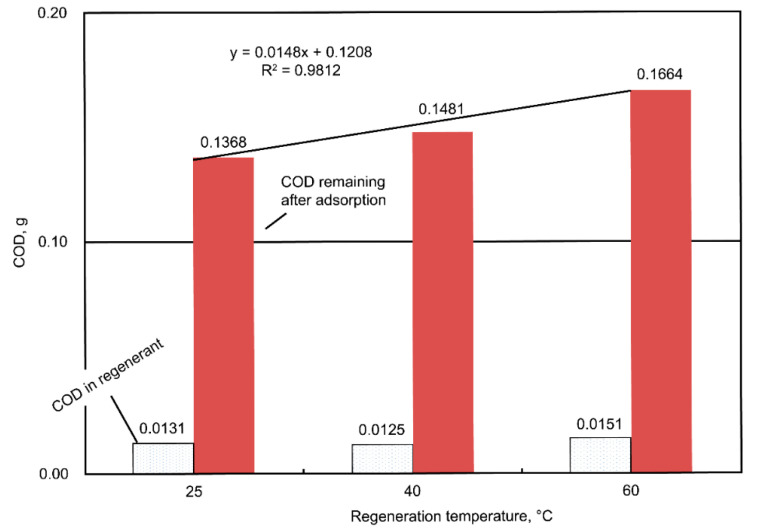
Plots of the COD residues (by weight) in the regenerant and the amounts of remaining COD in the AC-treated wastewater under various regenerating temperatures and a fixed pH value of 4.7. The lower the regeneration temperature, the less COD remained after adsorption.

A similar test to determine the most applicable dose of the iron complex was also conducted. The highest percent COD removal was noted at an iron-complex dosage of 2.12 mmole/dm^3^ (Figure 5). Furthermore, the effects of pH and temperature during the regeneration are shown in Figure 6 and Figure 7, respectively. The pH variation had non-obvious effects on the COD adsorption by the regenerated AC (Figure 6). The total COD input was 0.5407 g in 60 mL (i.e., initial concentration = 9012 mg COD/dm^3^) of the dyeing wastewater, and the amount of the unadsorbed COD ranged from 0.1368 to 0.1500 g (i.e., averaged value = 0.1431 ± 0.0028 g, which was equivalent to 26.5% of the COD unadsorbed); meanwhile, the COD in the regenerant ranged from 0.0220 to 0.0292 g (i.e., averaged value = 0.0252 ± 0.0015 g, which was equivalent to 4.7% of the COD unadsorbed). From the point of view of the mass balance, roughly 68.8% (= 100% − 26.5% − 4.7%) of the COD was considered oxidized during the regeneration stage. As for the temperature effect, higher regeneration temperatures were associated with more unadsorbed COD (Figure 7). At a regeneration temperature of 60 °C, 30% less COD was adsorbed after AC regeneration than at ambient temperature, which could be caused by the rather vigorous oxidative activities at a higher temperature, resulting in AC’s micro-pore destruction or blockage by certain oxidized intermediates. Similar mass balance results were also computed, which indicated that there was an oxidation of the adsorbed dyes (~70% of the total COD input) during the regeneration stage.

Herein, the collective results suggested the following: (1) more than 50% COD adsorption in 24 h by the used AC was doable at a wastewater-to-AC ratio of 12 mL/g-AC; (2) suitable adsorption conditions were low pH, and AC regeneration conditions were low temperature; and (3) ~70% of the COD was oxidized in 1 h during the regeneration. Thus, a lab-scale consecutive adsorption and regeneration run looked promising and was conducted.

**Figure 8:**
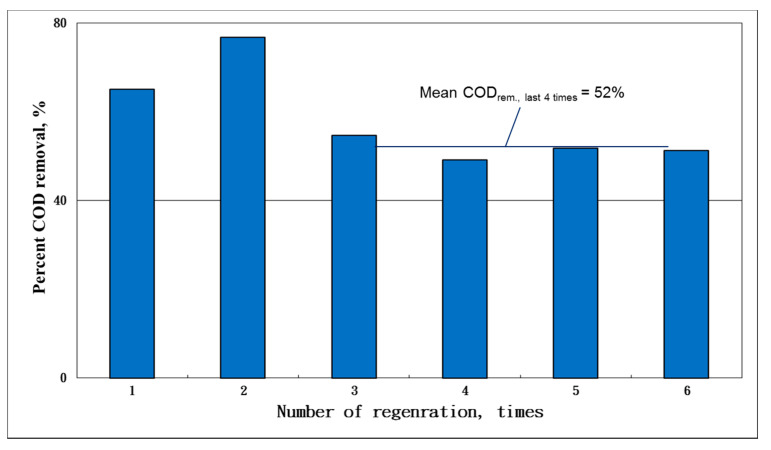
Plots of the percent COD removed versus the number of times AC was regenerated. The results showed that, at the targeted 50% adsorption of the COD, the reuses of the generated ACs seemed doable.

**Figure 9:**
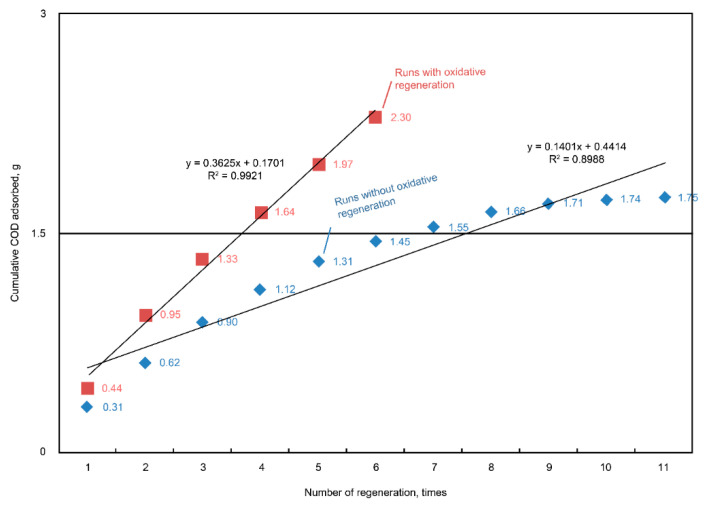
Plots of the cumulative COD adsorbed over the consecutive daily operations of AC adsorption and regeneration. The results showed a linear increase in terms of the cumulative COD adsorption during each adsorption–oxidation cycle.

The results of the consecutive adsorption–oxidation operation showed that more than 50% of the COD was consistently achieved with a 52% removal of the last four adsorption runs (Figure 8). By comparison with the cumulative COD adsorption between the regeneration and non-regeneration runs (11 cycles), we found that 5 g of regenerated AC could adsorb 0.3625 g-COD per cycle in the regeneration cases (Figure 9). On average, the adsorption capacity (q) value of the unit AC in each adsorption step was equal to 0.0725 g-COD/g-AC. Based on the previous material balance results, the adsorbed COD was presumably oxidized, so that the pore vacancy could be used again to uptake the COD during the next cycle.

**Figure 10:**
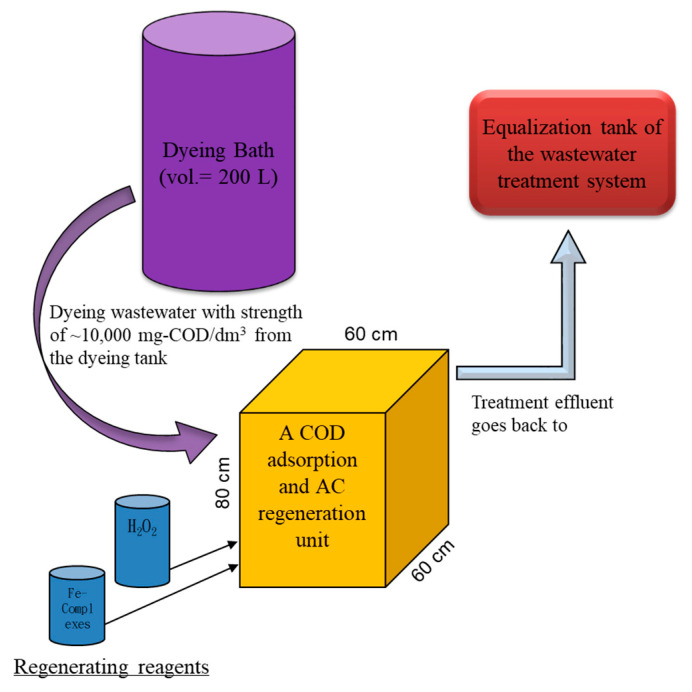
A proposed layout of the two-step treatment unit in treating a high-COD dyeing effluent directly from a 200 L dyeing bath. The designed unit can handle the high-COD wastewater drained from the 200 L dyeing bath and starts its adsorption–oxidation cycle to remove the COD in the wastewater.

Based on the obtained data and the current yarn dyeing process, the treatment of the high COD dyeing wastewater from a commonly used a 200 L dyeing bath using the proposed two-step treatment system looks applicable and was proposed with dimensions, as shown in Figure 10. To our knowledge, there is no such kind of the proposed setup in current operating dyeing facilities. The designed volume (i.e., 200 L) is sufficient to contain a batch effluent from the dyeing tank, so that the high COD wastewater can go directly to adsorption and regeneration in the proposed unit. Based on the results, a 50–60% removal of the COD would greatly minimize the COD flowing back to the equalization tank, so that the overloading scenarios of the existing in-plant biological treatment units could be avoided. To operate the proposed unit, first, a certain amount of the high COD wastewater from the dyeing tank is to be directed to a reactive unit filled with designated AC (20 kg if 120 L of wastewater are treated) for 24 h adsorption. Since the results suggested that the dyeing bath effluent COD was adsorbed more at acidic pH, regardless of temperature variation, neither pH nor temperature needs to be adjusted at this stage.

Second, the treated wastewater is to be screened and drained to the head (i.e., equalization tank) of its wastewater treatment facility after 24 h. Third, EDTA–Fe and hydrogen peroxide at initial concentrations of 2.12 mmole/dm^3^ and 2.5%, respectively, are to be added in series to start the AC regeneration for 1 h. Both Du and Chen [24] and mass balance results confirmed the degradation of adsorbed COD. Since the regeneration of AC could be greatly performed with minimal effects of pH and at ambient temperature during oxidation, no adjustment of either factor is needed. Finally, the regenerant is to be screened and drained to the head of its wastewater treatment facility as well, and a new cycle begins. According to consecutive operation, the applicability of the two-step processes in treating the high-COD dyeing wastewater looks promising. However, we do notice that the current application of this technique could be limited to the dyeing bath design and its operations. The highest COD effluent of a dyeing bath happens at its first discharge of the dyeing process, but for the most available dyeing bath operation, a separation of each batch effluent according to various COD strengths is not applicable. Some retrofits of the dyeing units or dyeing processes are considered necessary.

## 3. Material and Methods

High-COD dyeing wastewater was collected directly from some textile dyeing companies in Tainan City, Taiwan, and its COD content, pH, and suspended solids (SSs) were measured by standard methods [27]. Analytical results are given in Table 2. AC was purchased from Sigma-Aldrich (St. Louis, MO, USA) as 20–60 mesh granules. A pre-test of the purchased AC showed it followed the Langmuir isotherm adsorption (data not shown). H_2_O_2_ (*w*:*w*: 30%) was also purchased from Sigma-Aldrich (USA) and served as an oxidant in the regenerative reactions. The iron complex, for catalyzing the regeneration procedure, was made with EDTA and FeSO_4_ from Mallinckrodt Baker, Inc. (Phillipsburg, NJ, USA) at a 1:1 molar ratio. The COD samples were analyzed by the NIEA W515.55A standard method announced by the Taiwan Environmental Protection Agency [28]. After adding the samples to the pre-prepared COD reagent, a ROCKER CR-25 (Rocker Scientific Co., Ltd., Taipei City, Taiwan) was used for thermo-lysis, and this was followed by direct COD readings with a HACH DR/890 colorimeter (Hach Co., Ltd., Loveland, CO, USA).

**Adsorption and regeneration operations**—For adsorption tests, 125 mL flasks were used as reaction units. Each flask contained 60 mL of the dyeing wastewater, and the designated amounts of AC. Flasks were then stirring-mixed for 24 h and were sampled for measuring residual COD at designated times. For AC regeneration, 10 mL of peroxide and 0.2 mL of the Fe–EDTA reagent were added at the designated dosages, and the samples were allowed to react (i.e., being regenerated) for 1 h. Then, the COD of the regenerant was determined.

**Determination of AC’s adsorption capacity**—For testing the COD adsorption capacity of the used AC, the following two runs were conducted: one run used 0.1 to 5 g AC for adsorbing COD from 60 mL dyeing wastewater, so that the AC’s linear saturated adsorption capacity (q) was determined; the other compared the separate adsorption curves for one spike or multiple spikes of 60 mL dyeing wastewater with 5 g of the AC to determine whether 50% or more of COD was adsorbed.

**Determination of regeneration conditions**—To achieve the most applicable AC regeneration conditions, the virgin AC was first saturated with 60 mL of the dyeing wastewater, and then, initial doses of peroxide of 0–30% were added to initiate oxidative regeneration at an initial iron-complex concentration of 2.12 mmole/dm^3^. The regenerated AC was used to adsorb the dyeing wastewater again, and its adsorption capacity with respect to the virgin AC was given. Similarly, saturated AC was regenerated at 0.125% of peroxide and 0.8–4.15 mmole/dm^3^ of EDTA–Fe complex conditions. The COD adsorption after AC regeneration was also examined.

**Factor analysis**—To examine the effect of pH and temperature during AC adsorption and regeneration operations, pH values in the range of 4–10 and temperatures in the range of 25–60 °C were used. Wastewater pH was adjusted by 1 M of H_2_SO_4_ or NaOH as needed. A strong acidic or basic pH (i.e., less than 4 or greater than 10) was not preferable due to the costs and the inconvenience while adjusting the pH in the wastewater stream. The temperature was controlled by a temperature-adjustable oven (Model S300S; FIRSTEK Co., Shenzhen, China).

**Consecutive adsorption and oxidation test**—To confirm the utility of the adsorption and the regeneration cycle for treating high-COD dyeing wastewater, a series of five consecutive runs of the adsorption–oxidation operation were conducted at the obtained most applicable adsorption and oxidative conditions. The percent COD removal of the treated wastewater in each cycle was examined, so was the cumulative COD adsorbed in each cycle.

To implement quality assurance procedures, each measurement was tested three times ahead to calculate the percent relative standard deviation (%RSD). The %RSD was calculated by dividing the standard deviation with the mean value of the measurements multiplied by 100%, and a maximum limit of 5% was allowed to ensure each measurement was trustworthy. During the experimental period, each sample was replicated with some even quadruplicated, and the results were summarized and presented as average values.

## 4. Conclusions

The use of 5 g of AC was deemed sufficient to adsorb 50–60% of the COD from 60 mL of the dispersed dye-enriched wastewater. The adsorption capacity (q_max_) of the AC used was about 0.165 g-COD/g-AC. No adjustments to pH or temperature were necessary during COD adsorption operations using AC. To regenerate COD-saturated AC, reagents including peroxide at a concentration of 2.5% and an iron complex at a concentration of 2.12 mmole/dm^3^ were deemed sufficient. Under such conditions, COD adsorption increased by ~7% after the first regeneration, and it dropped by ~10% otherwise. The results suggested that during the regeneration operation, a larger pore surface was exposed. In addition, pH had little effect on AC regeneration, but higher temperatures resulted in lower AC adsorption after regeneration. When the regeneration temperature was maintained at 25 °C, about 30% higher COD adsorption was observed than that at 60 °C. During five executive adsorption and regeneration cycles, about 0.3625 g-COD/5-g AC were removed per cycle, which equaled to q = 0.3625/5 = 0.0725 g-COD/g-AC. These results demonstrated that the adsorbed COD was oxidized during regeneration operation. Overall, the use of AC adsorption to concentrate the COD of dyeing wastewater, followed by EDTA–Fe catalyzed, the peroxide-oxidized treatment of saturated AC was likely a promising alternative to remove high-COD dyeing wastewater with a much less oxidant and higher COD treatment efficiency. A scale-up, 200 L adsorption–regeneration unit was proposed, and its operational processes and potential limits were addressed.

## Figures and Tables

**Table 1:** Examples of commonly used dyes in the studied dyeing company, including acid, reactive, and disperse dyes.

Type	Properties	Clothes to Dye	Major Structure	Ligand	Target Compound
Acid dyes	Water-soluble; used at low pH conditions; easy to use; and forming an ionic bond	Wool, silk, nylon, and polyamide fibers	Azo: 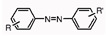 Anthraquinone: 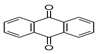 Arylmethane: 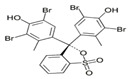	–SO_3_H –COOH –OH	Acid Red 114 (solubility = 60 g/dm^3^) 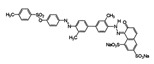
Reactive dyes	Water-soluble; easy to use; potential to be hydrolyzed; forming a covalent bond	Cotton, blended fibers, wool, and silk	Azo, Anthraquinone and phthalocyanine: 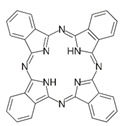	–SO_3_Na –RSO_2_R –C_4_H_4_N_2_	Reactive Black 5 (solubility = 60 g/dm^3^) 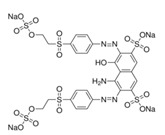
Disperse dyes	Minimal solubility; added with dispersive chemicals	Polyester/acrylic/polyamide fibers, cellulose acetate, and nylon	Azo and anthraquinone	–OH –R-C-O-O-R –R-O-R –CN –NH_2_ –NO_2_	Disperse Black EX-SF (minimal solubility) 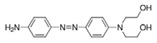

**Table 2:** Characteristics of the studied dyeing wastewater with the sampling time and some statistics.

Property	No. of Sample	Range	Average ± Std. Dev. (%RSD *)
Chemical oxygen demand (COD; mg/dm^3^)	18	9160–10,503	9507 ± 320 (3.4%)
pH	12	4.45–4.90	4.67 ± 0.1 (3.1%)
Suspended solid (SS; mg/dm^3^)	6	380–420	402 ± 16.06 (4.0%)

* %RSD = percent relative standard deviation = (std. dev./mean) × 100%. Where, std. dev. means standard devidation.

## Data Availability

Not applicable.
